# Whole‐exome sequencing reveals novel *USP9X* variant in female fetus with isolated agenesis of the corpus callosum

**DOI:** 10.1002/ccr3.2051

**Published:** 2019-02-19

**Authors:** Jerica L. Lenberg, Dolores H. Pretorius, Eric S. Rupe, Marilyn C. Jones, Gladys A. Ramos, Tara S. Andreasen

**Affiliations:** ^1^ Department of Genetic Counseling Augustana University Sioux Falls South Dakota; ^2^ Department of Radiology University of California San Diego California; ^3^ Division of Genetics, Department of Pediatrics University of California San Diego California; ^4^ Division of Maternal‐Fetal Medicine, Department of Reproductive Medicine University of California San Diego California

## Abstract

Whole‐exome sequencing in a female fetus detected a *USP9X* variant. This X‐linked gene was recently associated with intellectual disability and distinct pattern of malformation in females. Isolated agenesis of the corpus callosum has not been reported in association with *USP9X*. Identifying this variant impacted management of the subsequent pregnancy.

## 1 INTRODUCTION

Variants in the ubiquitin‐specific protease 9X (*USP9X)* gene have been associated with X‐linked syndromic intellectual disability. *USP9X* is required for neural cell migration and brain development in early embryogenesis. In males, hemizygous hypomorphic variants can cause syndromic intellectual disability while loss‐of‐function variants are believed to be lethal. Females with hypomorphic variants are typically unaffected. However,* USP9X* loss‐of‐function variants in females have recently been identified to cause a recognizable pattern of malformation and intellectual disability. Additionally, over half of described females with loss‐of‐function variants in *USP9X* have a hypoplastic corpus callosum. Here, we describe a female fetus with isolated complete agenesis of the corpus callosum and a likely pathogenic variant in *USP9X* detected by whole‐exome sequencing. This report describes a novel de novo presumed loss‐of‐function variant resulting in an X‐linked dominant condition in a female fetus. Finally, this report highlights an emerging role of whole‐exome sequencing in cases of ultrasound anomalies following normal chromosomal microarray.

Intellectual disability of genetic etiology has been associated with nearly a hundred different genes and is implicated in all patterns of inheritance. Variants in the ubiquitin‐specific protease 9X (*USP9X)* gene have been associated with X‐linked syndromic intellectual disability. *USP9X* encodes for a highly conserved deubiquitinase enzyme which plays a role in protein trafficking, cell polarity, cell death, and consequently influences neural cell migration, formation of the hippocampal dentate gyrus, ciliary function, tumor suppression, and oncogene pathways.^1^, ^2^, ^3^ Hemizygous hypomorphic variants in males can be associated with intellectual disability, short stature, hypotonia, behavioral issues, gastrointestinal problems, dysmorphic features, and a predisposition to seizures.^4^, ^5^ Hypomorphic variants in *USP9X* most often result in affected males and unaffected carrier females.

However, females with loss‐of‐function variants in *USP9X* have recently been described as having a syndromic pattern of malformation including intellectual disability, dysmorphic facial features, severe congenital malformations, and brain abnormalities.^6^, ^7^, ^8^ The mechanism is likely in part because *USP9X* at least partially escapes X‐inactivation.^8^, ^9^, ^10^ In vitro studies show loss‐of‐function variants in *USP9X* cause reduction in axonal growth and neuronal cell migration in the brain.^4^, ^8^ The severity of the phenotype in females is suggested to be correlated with the extent of *USP9X* truncation.

In contrast, loss‐of‐function variants have not been reported in affected males nor in the population database Exome Aggregation Consortium (ExAC) suggesting hemizygous loss‐of‐function variants in *USP9X *in males cause prenatal or neonatal demise.^4^, ^8^ *USP9X *is required for neural development and in vivo studies reveal whole‐brain depletion of *USP9X* causes postnatal lethality in male mice.^2^, ^3^

Here, we describe a female fetus presenting with complete agenesis of the corpus callosum and was found to have a novel de novo likely pathogenic variant in *USP9X*.

## 2 CLINICAL REPORT

**Figure 1:**
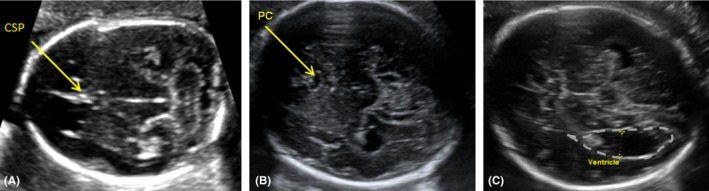
Axial views of head and brain using fetal ultrasound. A, At 18w3d gestation, an interhemispheric fluid collection is noted and was called a cavum septi pellucidi (CSP). No abnormal findings were documented. B, At 29w6d gestation, an interhemispheric fluid collection is again seen and now noted to have abnormal morphology consistent with a pseudocavum (PC). Additionally noted at this time (but not pictured) were teardrop‐shaped ventricles consistent with colpocephaly. C, At 31w2d gestation, the enlarged and tear‐dropped shaped bilateral lateral ventricles (left ventricle is depicted with dashed line) consistent with colpocephaly were again seen

**Figure 2:**
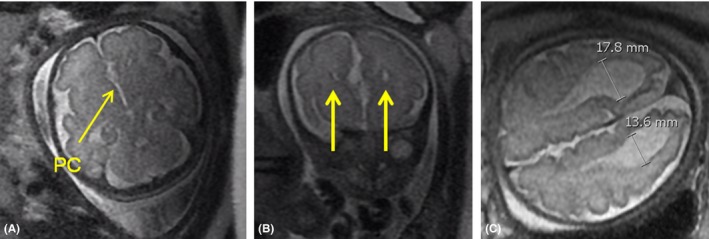
Fetal MRI at 31w3d gestation (single shot fast spin‐echo T2‐weighted images). The corpus callosum is not visualized on any images. A, Axial image demonstrates an abnormal interhemispheric fluid collection anteriorly which does not have morphology consistent with cavum septi pellucidi (CSP) and is therefore a pseudocavum (PC). B, Coronal image demonstrates trident‐shaped frontal horns of the lateral ventricles (arrows). C, Axial image shows dilation of the occipital horns of the lateral ventricles with teardrop configuration consistent with colpocephaly

The patient was a 28‐year‐old G1P0 female of Caucasian and Filipino ancestry, and the patient's partner (father of the baby) was a 28‐year‐old male of Northern European ancestry. The first‐trimester scan at 7w5d showed a singleton intrauterine pregnancy. The anatomy scan at 18w3d was unremarkable with brain and head imaging showing interhemispheric fluid collection and what was identified to be a normal cavum septi pellucidi (Figure 1A). A nonroutine growth ultrasound was performed at 29w6d gestation after the patient expressed she had a feeling something was wrong and requested additional imaging. This ultrasound revealed bilateral borderline ventriculomegaly measuring 11 mm on the left and 12 mm on the right. Irregular interhemispheric fluid collection was again visualized, consistent with a pseudocavum, and abnormal morphology of the lateral ventricles consistent with colpocephaly was present (Figure 1B). At this time, fetal growth was in the 28th percentile for gestational age and appropriate interval growth was noted when compared to prior ultrasound. A fetal MRI was recommended to allow for further characterization of the intracranial structures. An additional fetal brain ultrasound was performed at 31w2d when the patient presented with preterm uterine contractions (Figure 1C). A fetal brain MRI at 31w3d revealed the absence of the corpus callosum on all images, morphology consistent with a pseudocavum (Figure 2A), trident‐shaped frontal horns (Figure 2B), and a colpocephalic configuration of the lateral ventricles with bilateral ventriculomegaly measuring 13.6 mm on the left and 17.8 mm on the right (Figure 2C). The MRI was otherwise unremarkable.

Until this point, the patient's negative genetic testing included noninvasive prenatal testing (NIPT) and sequential integrated screening. Based on NIPT results, the risks for select aneuploidies were <1/10 000 for Down syndrome, Trisomy 18, and Trisomy 13. Carrier screening showed the patient to be a carrier of pseudocholinesterase deficiency and Pendred syndrome, and her partner to be a carrier for phenylalanine hydroxylase deficiency (PKU), but neither were carriers for both. The patient and her partner did not have personal or family history thought to be contributory including hereditary conditions, brain abnormalities, intellectual disability, stillbirths, or two or more pregnancy losses. The patient denied medication use, illicit drug use, or exposure to infectious disease. Congenital infection workup including toxoplasmosis, rubella, cytomegalovirus and herpes virus (TORCH) titers was negative for an acute infection.

The couple was counseled on management options including amniocentesis for karyotype and chromosomal microarray, pregnancy termination with autopsy and/or chromosomal microarray, or continuation of pregnancy with postnatal assessment. The couple elected for termination of the pregnancy at 33w6d with autopsy and chromosomal microarray. The fetal autopsy revealed extensive maceration and interhemispheric cortex with radial gyri consistent with agenesis of the corpus callosum. There were some abnormalities of the placenta which included small immature placenta, hyper‐twisted umbilical cord, intervillous thrombus, areas of increased syncytial knots, foci of avascular villi, and a three‐vessel cord. The autopsy did not reveal any additional abnormalities, and the chromosomal SNP microarray performed using DNA extracted from fetal skin was normal.

Given the normal testing thus far, and the inability for chromosomal microarray to detect sequencing errors, whole‐exome sequencing was desired by the couple. Trio whole‐exome sequencing was performed at ARUP Laboratory using DNA from fetal skin and blood samples from both parents and phenotype‐driven analysis. Whole‐exome sequencing with Sanger sequencing confirmation detected a likely pathogenic de novo variant in the fetus within the *USP9X* gene (c.6944C>T; p.Ser2315Phe; NM_001039590.2; g.41084187 (GRCh37)). This missense variant had not been reported in the literature, was not listed in any gene‐specific databases, and had not been previously identified by the performing laboratory. Additionally, the laboratory reported this variant is absent from the population databases 1000 genomes, NHLBI GO Exome Sequencing Project (ESP), and the Genome Aggregation Database (gnomAD). Two of three in silico prediction tools predicted the variant to be damaging, while one predicted it to be benign (SIFT: damaging; Mutation Taster: disease causing; PolyPhen2: benign). An X‐inactivation assay determined the fetal sample to have a pattern of 30:70 skewing which is within normal limits. Thus, the performing laboratory determined the c.6944C>T variant to be likely pathogenic and the likely cause of the fetus's phenotype.

While awaiting results from the whole‐exome sequencing, the couple conceived another pregnancy. The *USP9X* variant in the proband was de novo but parental germ line mosaicism could not be ruled out. The second pregnancy has thus far had normal ultrasound scans and tested negative for the *USP9X* likely pathogenic variant using prenatal targeted testing.

## 3 DISCUSSION

We describe a female with a likely pathogenic variant in *USP9X* presenting with complete agenesis of the corpus callosum, bilateral ventriculomegaly, and an abnormal gyration pattern of the frontal lobe.

During routine prenatal ultrasound, the fetal brain is interrogated for various abnormalities. Structures required to be documented as normal during 18‐week routine screening ultrasound examination by the American Institute of Ultrasound in Medicine, American College of Obstetrics and Gynecology, American College of Radiology, and the Society of Radiologists in Ultrasound include the cavum septi pellucidi, the lateral cerebral ventricles, choroid plexus, midline falx, cerebellum, and cisterna magna. The presence of the cavum septi pellucidi is of utmost importance in excluding complete agenesis of the corpus callosum. Additional findings that could signal the complete or partial agenesis of the corpus callosum include the presence of a pseudocavum, dilation and elevation of the third ventricle, teardrop‐shaped lateral ventricles known as colpocephaly (on axial images), trident‐shaped frontal horns of the lateral ventricles (on coronal images), presence of Probst bundles medial to the frontal horns causing the trident shape configuration, radiating sulci rather than normal parallel sulci (on sagittal images), or absence of the pericallosal artery. Sometimes the corpus callosum is not seen on sagittal images; however, in partial agenesis of the corpus callosum only the posterior portion is absent. Direct visualization of the normal corpus callosum is technically challenging in many patients, but especially before 22 weeks.

Agenesis of the corpus callosum is one of the most common birth defects yet prognosis remains difficult to predict and relies heavily on empirical data. The corpus callosum is a large collection of nerves that enables transfer of information between the right and left hemisphere of the brain. Isolated agenesis of the corpus callosum does not impact intelligence in most cases. Roughly, 65%‐75% of individuals with agenesis of the corpus callosum have average intelligence quotients, while around 12% of individuals have severe intellectual disability, and the remaining 13%‐23% of individuals have moderate intellectual disability.^11^, ^12^, ^13^ These potential outcomes and the possibility of a syndromic etiology were provided to the patient and her partner during genetic counseling and perinatal consultations before identifying the *USP9X *variant. In contrast, available literature suggests 100% of females with loss‐of‐function variants in *USP9X* have intellectual disability or developmental delay.^6^, ^7^, ^8^ The presence of this variant implicated a drastically different prognosis and recurrence risk. Although whole‐exome sequencing was performed after termination, the information available to the family for decision‐making was significantly altered.

The fetus did not have dysmorphic features or other congenital anomalies described in females with loss‐of‐function variants in *USP9X*. However, most described females have brain abnormalities including hypoplastic corpus callosum (62%), asymmetric enlarged ventricles (73%), and abnormal gyration pattern of the frontal lobes (50%).^8^ It is possible the c.6944C>T variant does not cause complete loss‐of‐function of *USP9X* and permits some production of functional protein resulting in a milder phenotype. This mechanism is suggested by Au et al^6^ who describe a female with a variant in the 5′ untranslated region of *USP9X* with a phenotype of developmental delay, hypopigmentation, hyperpigmentation, joint hypermobility, and mild hypotonia, but with no dysmorphic features or congenital anomalies. The functional data on loss‐of‐function variants are limited to one study which showed fibroblasts and lymphoblastoid cells derived from females with loss‐of‐function variants in *USP9X* have reduced mRNA expression (n = 4) and protein levels (n = 1) compared to control females.^8^ There is a paucity of data on the c.6944T>C variant; however, the absence from all population databases, absence in males, lack of X‐inactivation skewing, in silico predictions, and the patient's phenotype suggest this variant causes a loss or reduction of *USP9X* function. Identifying less severely affected individuals helps establish the spectrum of a syndrome which is essential for recently characterized syndromes that may be more subject to ascertainment bias.

Lastly, the utility of whole‐exome sequencing for agenesis of the corpus callosum is not well‐characterized nor considered to be high‐yield for patients with structural abnormalities. Quantitative fluorescent PCR, karyotyping, and chromosomal microarray together identify the genetic etiology in around 40% of structurally abnormal fetuses—which leaves the etiology in around 60% of cases undetermined.^14^ A meta‐analysis found that 4.81% of prenatally diagnosed cases of complete agenesis of the corpus callosum had chromosomal abnormalities.^15^ When the etiology is not explained by chromosome abnormalities, the utility of whole‐exome sequencing is variable. A review of 31 studies found whole‐exome sequencing detects the likely cause of ultrasound anomalies in 6.2%‐80% of cases.^14^ This wide range is due to ascertainment method, trio or singleton testing, and whether multiple anomalies are present. Another study completed whole‐exome sequencing on 84 deceased fetuses with ultrasound anomalies ascertained after fetal demise or termination.^16^ Whole‐exome sequencing detected a positive result in 20% of fetuses overall, and in 25.8% of fetuses with central nervous system anomalies. More specific to agenesis of the corpus callosum, a retrospective study of 16 cases of prenatally identified agenesis of the corpus callosum showed four individuals underwent whole‐exome sequencing and two had positive results, yielding a diagnostic rate of 12.5%‐50%.^17^ Importantly, after postnatal evaluation many of the individuals in this cohort were shown to have other anomalies in addition to agenesis of the corpus callosum. Larger prospective studies are needed to evaluate the utility of whole‐exome sequencing in cases of true isolated agenesis of the corpus callosum. Nevertheless, this case helps establish the spectrum of phenotypes associated with *USP9X *variants, highlights the potential for X‐linked conditions to occur in heterozygous females, and supports the emerging role of whole‐exome sequencing after normal chromosomal microarray in cases of prenatal isolated agenesis of the corpus callosum.

## CONFLICT OF INTEREST

None declared.

## AUTHOR CONTRIBUTIONS

JLL: wrote the manuscript, designed the study, acquired data, analyzed data, and interpreted data. TSA: conceived and designed the study. DHP, ESR, MCJ, GAR and TSA: engaged in clinical care, acquired data, analyzed date, interpreted data, made critical revision for intellectual content, and approved the final manuscript.
